# Post-weaning Exposure to High-Fat Diet Induces Kidney Lipid Accumulation and Function Impairment in Adult Rats

**DOI:** 10.3389/fnut.2019.00060

**Published:** 2019-05-03

**Authors:** Cynthia R. Muller, Ana Paula O. Leite, Rodrigo Yokota, Renata O. Pereira, Anna Laura V. Americo, Nilberto R. F. Nascimento, Fabiana S. Evangelista, Vera Farah, Manasses C. Fonteles, Patricia Fiorino

**Affiliations:** ^1^Renal, Cardiovascular and Metabolic Physiopharmacology Laboratory, Health and Biological Science Center, Mackenzie Presbyterian University, São Paulo, Brazil; ^2^Experimental Pathophysiology Department, Faculty of Medicine, University of São Paulo, São Paulo, Brazil; ^3^Department of Medicine, Renal Division, Federal University of São Paulo, São Paulo, Brazil; ^4^Department of Medicine, Translational Medicine Division, Federal University of São Paulo, São Paulo, Brazil; ^5^Superior Institute of Biomedical Sciences, Ceara State University, Fortaleza, Brazil; ^6^School of Arts, Science and Humanities, University of Sao Paulo, São Paulo, Brazil

**Keywords:** post-weaning, high-fat diet, kidney lipid deposition, lipotoxicity, inflammation

## Abstract

**Aim:** We investigated the kidney morphofunctional consequences of high-fat diet intake since post-weaning in adult rats.

**Main Methods:** Male Wistar rats were divided into two groups: ND (normal diet; *n* = 10) and HD (high-fat diet; *n* = 10). The high-fat diet was introduced post-weaned and animals were followed for 8 weeks.

**Key Findings:** HD group did not change body weight gain even though food consumption has decreased with no changes in caloric consumption. The HD group showed glucose intolerance and insulin resistance. The glomerular filtration rate (GFR) was decreased *in vivo* (ND: 2.8 ± 1.01; HD: 1.1 ± 0.14 ml/min) and in the isolated perfusion method (34% of decrease). Renal histological analysis showed a retraction in glomeruli and an increase in kidney lipid deposition (ND: 1.5 ± 0.17 HD: 5.9 ± 0.06%). Furthermore, the high-fat diet consumption increased the pro-inflammatory cytokines IL-6 (ND: 1,276 ± 203; HD: 1,982 ± 47 pg/mL/mg) and IL-1b (ND: 97 ± 12 HD: 133 ± 5 pg/mL/mg) without changing anti-inflammatory cytokine IL-10.

**Significance:** Our study provides evidence that high-fat diet consumption leads to renal lipid accumulation, increases inflammatory cytokines, induces glomeruli retraction, and renal dysfunction. These damages observed in the kidney could be associated with an increased risk to advanced CKD in adulthood suggesting that reduction of high-fat ingestion during an early period of life can prevent metabolic disturbances and renal lipotoxicity.

## Introduction

High-fat diets are becoming increasingly common in many countries and they contribute to the development of chronic non-communicable diseases (NCDs), such as obesity, hypertension, and chronic kidney disease (CKD) (1). NCDs kill 41 million people each year, 71% of the world's total deaths (2). It is recommended that fats account for 20–35% of total energy intake (3), but daily total fat consumption accounts for 50% of total energy intake in some countries (4).

High-fat diets, in general, are associated with metabolic disorders, and the type of dietary fat is a determinant risk factor since saturated fats are more linked to a positive fat balance and visceral adipose tissue accumulation than to other types of fat (5). Saturated fat intake is also more associated with increased serum LDL and total cholesterol than the consumption of other fatty acids (6). The World Health Organization recommends a reduction in saturated fat consumption as one of the worldwide strategies to reduce mortality from chronic NCDs (7).

Children are important targets for food and beverage companies that use aggressive advertising strategies to generate a preference for diets with high levels of fat. As a result, the consumption of high-fat diets typically starts early in life, especially in developed and developing countries (8). High-fat diet habits in childhood can predict the development of several diseases in adulthood, such as obesity, hypertension, metabolic syndrome, and CKD (9, 10). Our group demonstrated that high-fat diet ingestion since the early period of life increases white visceral adipose tissue and induces cardiometabolic damage in adult rats (11). Te Morenga et al. (12) showed that a reduction of saturated fat intake was associated with significant reductions in LDL and total cholesterol and arterial blood pressure of children and adolescents aged 2–19 years old (12).

High-fat diet affects the energy balance (13) leading to lipid accumulation in ectopic sites and in intracellular compartments (14, 15). The renal ectopic accumulation of lipids associated with insulin resistance has been correlated with a progressive decline in renal function (14, 16, 17). The deleterious effects exerted by lipids on cells and tissues is called lipotoxicity (18, 19).

Many studies have demonstrated the link between altered lipid metabolism and the development of kidney injury in mice fed a high-fat diet (14, 17, 20, 21). The literature reports an important association between renal lipid accumulation and increased renal pro-inflammatory mediators, such as interleukin-1 (IL-1), interleukin-6 (IL-6), and tumor necrosis factor alpha (TNFα) (19). Furthermore, excessive renal lipid deposition can lead to renal tubular cell injury (22), tubulointerstitial fibrosis (23), podocytes damage, mesangial sclerosis (24), and structural glomeruli alterations (25, 26). Renal lipotoxicity is also strongly associated with the development of proteinuria, glomerulonephritis, and CKD (27). However, the role of renal lipid accumulation that could lead to kidney damage in a high-fat diet has not been completely understood.

Although the pathological consequences of a high-fat diet on the kidneys are well-documented, the repercussions in renal morphology and function as a result of a high-fat diet from an early age are not clear. Thus, the aim of this study was to investigate the effects of high-fat diet intake from weaning on the morphology and renal function of adult rats. We hypothesized that the intra-renal lipid accumulation induced by high-fat diet can be associated with damages in the renal morphology and function, leading to a higher risk of developing CKD in adulthood.

## Materials and Methods

### General Procedures

Experiments were performed in male Wistar rats post-weaned (21 days old), weighing between 50 and 60 g. The animals were randomly assigned to two groups and followed for 8 weeks: standard normal diet (ND, *n* = 10) and high-fat diet (HD, *n* = 10). The high-fat diet produced contained 30% of fat, 23% of carbohydrates and 19% of proteins. The fat in HD is composed mainly of saturated fat. The standard diet contained 3% of fat, 55% of carbohydrate and 22% of proteins (Nuvilab®, Paraná, Brazil). Caloric densities of a high-fat diet and standard diet were, respectively 381 and 257 kcal/100 g. Animals were maintained in the Central Animal Facility at the Mackenzie Presbyterian University (Sao Paulo) under the same housing conditions (12-h light/12-h dark cycle, temperature 23 ± 2°C) with free access to tap water and food *ad libitum*. All procedures were performed in accordance with the Guide for the Care and Use of Laboratory Animals published by the US National Institutes of Health (n. 85-23, revised in 1996) and approved by the Ethics Committee of Mackenzie Presbyterian University (Protocol: 063/02/2010). Body weight was measured weekly at the same time of the day using a digital balance (TOLEDO, model 9094c/4). Body weight gain was calculated as the difference between the body weight measured at the beginning and at the end of the protocol.

### Glucose Tolerance Test (GTT)

GTT was performed after 8-week protocol. After an 8-h fast, glucose (1.5 g/kg body weight) was injected as a bolus intraperitoneally. Blood glucose concentration was determined by using a glucometer (AccuChek Advantage Roche Diagnostics®). Blood samples were taken from a cut made on the tip of the tail at 0, 15, 30, 60, and 90 min after glucose administration. The area under the curve (AUC) was calculated using GraphPad Prism 5 (GraphPad Software Inc, San Diego, CA, EUA).

### Insulin Tolerance Test (ITT)

Seventy two hours after the GTT test, a similar procedure was performed for ITT. Briefly, after a 4-h fast, rats were anesthetized with Pentobarbital (50 mg/kg body weight, *i.p*.) and an insulin load (0.75 U/kg body weight) was injected as a *bolus* in the caudal vein. Blood glucose levels were determined from a cut made on the tip of the tail at 0, 4, 8, 12, and 16 min after insulin administration. Constant rate for blood glucose disappearance (Kitt) was calculated using the formula 0.693/t1/2, and the blood glucose half-time (t1/2) was calculated from the slope of the least squares regression of the blood glucose concentration during the linear phase of decline (11, 28).

### Kidney Function Evaluation

In the 7th week of the protocol, the animals were housed individually in metabolic cages (Tecniplast, Buguggiate, VA, Italy). Urine samples were collected during the 24-h period and used to determine urine creatinine. A blood sample (500 μL) was also collected at the end of the 24 h-period. Urinary and serum creatinine levels were quantified using a colorimetric method (LABTEST Biochemical Kit, Brazil). The creatinine clearance was used to estimate the Glomerular Filtration Rate (GFR) and was calculated using the following formula: [(Urine (Creatinine) × Urine Vol)/Serum (Creatinine)].

### Isolated Perfused Kidney Method

At the end of the 8 weeks, rats were anesthetized with sodium pentobarbital (50 mg/kg body weight *i.p*.) and the right renal artery was cannulated through the mesenteric artery, without blood flow interruption, and placed into the perfusion system (29), isolating the kidney from endocrine and neural interference. The perfusate was a modified Krebs–Henseleit solution with the following composition (in mM): 147 Na^+^, 5 K^+^, 2.5 Ca^2+^, 2.0 Mg^2+^, 110 Cl^−^, 25 HCO^3−^, 1 SO4^2−^, and 1 PO4^3−^. This was dialyzed for 48 h after the addition of six grams of bovine serum albumin (BSA) (29). Immediately before starting the perfusion, 100 mg of glucose, 50 mg of urea, and 50 mg of inulin was added to the perfusate solution. The pH was then adjusted to 7.4 and the solution placed in the perfusion system. The perfused rat kidney model followed the technique previously described by Bowman and Maack and modified by Fonteles et al. (29, 30) by the introduction of a silastic membrane oxygenator into the perfusion line. Prior to each experiment, the system was calibrated for flow and resistance. Each experiment was divided into two periods of 30 min each, these sample collection periods were further subdivided into equal intervals of 10 min. During each 10-min period, aliquots of perfusate and urine were collected to determine creatinine, sodium, and potassium. The perfusion pressure (PP), renal vascular resistance (RVR), urinary flow (UF), GFR, and the percentage of tubular transport of sodium (%TNa^+^), potassium (%TK^+^), and chloride (%TCl^−^) were determined. The percent of proximal and distal tubular sodium, potassium, and chloride transport were calculated using free water and osmolar clearances as described originally by Martinez-Maldonado and Opava Stitzer (31).

### Histological Analysis

The left kidney was used for glomerular injury analysis in H&E stained (Sigma) sections of the kidney (5 μm) embedded in Paraplast. Digital images from thirty glomeruli per animal were obtained using a light microscope (Leica) at 400x magnification. After digitalization, Bowman's capsule area (BCA), glomerular tuft area (GTA), and Bowman's space area (BSA) were traced and calculated using a computerized morphometric analysis system (Image Pro-Plus 4.1; Media Cybernetics, Silver Spring, MD, USA).

Lipid content was measured using quantitative histochemistry of Oil Red O (Sigma-Aldrich) stained kidneys. Tissue sections (8 μm thickness) obtained in a cryostat were examined by light microscopy at 200x magnification and analyzed by a computerized morphometric analysis system (Image Pro-Plus 4.1; Media Cybernetics, Silver Spring, MD, USA). The slides were counterstained with hematoxylin to visualize the nuclei. Lipid accumulation was determined in 12 images per animal based on the percentage of area occupied by lipid droplets. Histological analyses were blinded conducted by RO Pereira.

### Cytokine Measurement

In a subgroup of randomly selected rats (ND: *n* = 6; HD: *n* = 6) cytokines were evaluated in the right kidney approved by the Ethics Committee of Mackenzie Presbyterian University (Protocol: 108/03/2014).

Measurement of cytokines was performed using the MILLIPLEX™ cytokine panel (Merck Millipore, Billerica, MA), a bead-based immunoassay which allowed the simultaneous quantification of the cytokines IL-1b, IL-6, TNF-α, and IL-10 in kidney samples. The results were normalized by kidney total protein.

### Statistical Analyses

The statistical analysis was performed by using GraphPad Prism 5. The results were analyzed using the unpaired Student's *t*-test. The data were reported as mean ± SEM. The *p*-value for significant differences was set at *p* ≤ 0.05.

## Results

### Body Weight and Food Consumption

No differences in body weight were observed between groups prior to or after the experimental protocol. In addition to this, no significant difference in body weight gain was found between groups. Although the food consumption (g/animal/24-h) was reduced in the HD group, there was no difference in caloric intake (Kcal/animal/24-h) when compared to the ND group (Table 1).

**Table 1:** Metabolic parameters at the end of the protocol of 8 weeks.

	**ND**	**HD**
Initial body weight (g)	47 ± 0.9	49 ± 0.8
Final body weight (g)	312 ± 7	295 ± 6.3
Weight gain (g)	265 ± 8	246 ± 6
Food consumption (g/animal/24 h)	26 ± 0.3	21 ± 0.7^*^
Caloric consumption (Kcal/animal/24 h)	79 ± 1	79 ± 2.7
Fasting glucose (mg/dL)	108 ± 5	99 ± 6
AUC (mg/dL/min)	144 ± 6	200 ± 9^*^
Kitt (mg/dL)	4 ± 0.2	3.3 ± 0.2^*^

*AUC, Area Under the Curve; Kitt, Rate constant for glucose disappearance; ND, Normal Diet; HD, High-Fat Diet*.

* *p ≤ 0.05 vs. ND*.

### Glucose Metabolism

As shown in Table 1, high-fat diet consumption did not change fasting glucose but promoted an increase in the AUC and a decrease in Kitt.

### Renal Function

**Figure 1:**
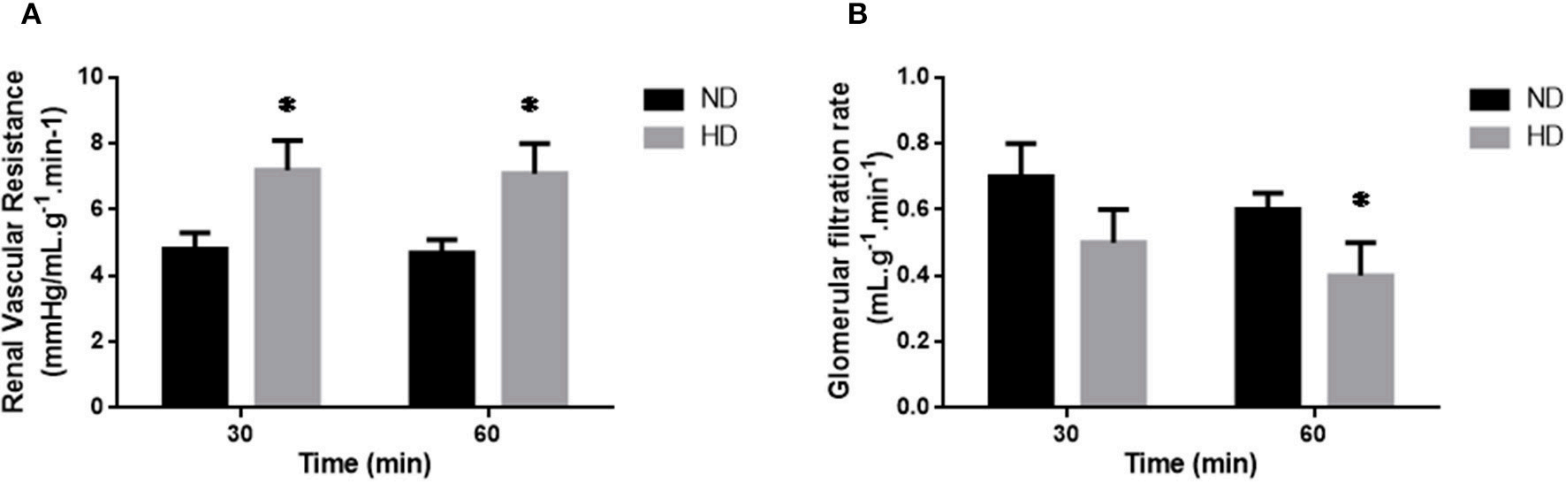
Isolated perfused kidney. Renal Vascular Resistance (**A**; RVR), and Glomerular Filtration Rate (**B**; GFR) of isolated perfused kidneys from ND, Normal Diet; HD, High-Fat Diet. The results are expressed as mean ± SEM *p ≤ 0.05 vs. ND.

Indices of kidney function are shown in Table 2 and Figure 1. There was a decrease in the *in vivo* GFR (ND: 1.8 ± 0.19; HD: 1.1 ± 0.14 ml/min), UF (ND: 9.8 ± 0.54 HD: 3.7 ± 0.19 mL/24-h) and water intake (ND: 26.9 ±1.45; HD: 16.75 ± 1.35 mL/24-h) in HD group when compared with ND. In addition, the serum creatinine was increased in HD (ND: 0.55 ± 0.071; HD: 0.71 ± 0.14 mg/dL).

**Table 2:** Renal functional parameters of Perfusion Pressure, Renal Plasmatic Flow, and tubular transport of Sodium, Potassium, and Chloride at the end of the protocol of 8 weeks of isolated perfused kidney method.

**Isolated perfused kidney parameters**	**ND**	**HD**	**ND**	**HD**
	**30 min**		**60 min**	
PP	114.7 ± 3.9	130.0 ± 6.7	112 ± 3.5	130 ± 7^*^
RPF	26 ± 2.1	19.2 ± 1.3^*^	26 ± 2	20 ± 1.4^*^
UF	0.14 ± 0.014	0.15 ± 0.03	0.14 ± 0.01	0.23 ± 0.04^*^
%T Na	83.6 ± 0.9	83.7 ± 3.2	80 ± 3	72 ± 5
%T K	61.3 ± 5.3	69.0 ± 5.6	61 ± 5	65 ± 3
%T Cl	89.9 ± 3.7	79.8 ± 4.0	81 ± 3	76 ± 4

*Perfusion Pressure (PP, mmHg), RPF (Renal Plasmatic Flow, mL.g^−1^.min^−1^), UF (Urinary Flow, mL.g^−1^.min^−1^), Percentage of Sodium tubular transport (%T Na^+^), Percentage of Potassium tubular transport (%T K^+^) and Percentage of Chloride Tubular transport (%T Cl^−^). ND, Normal Diet; HD, High-Fat Diet*.

* *p ≤ 0.05 vs. ND*.

In the isolated perfused kidney, the RVR was increased in both experimental periods, achieving 47% increase after 60 min perfusion, in the HD group (Figure 1A). UF was augmented at 60 min (77%) (Table 2). The GFR showed a decrease (34%) in the HD group at 60 min (Figure 1). Furthermore, the RPF decreased in both times, and the PP increased at 60 min, in HD. However, the tubular ion transport of Na+, Cl–, K+ have not changed in the HD (Table 2).

### Histological Analysis

**Figure 2:**
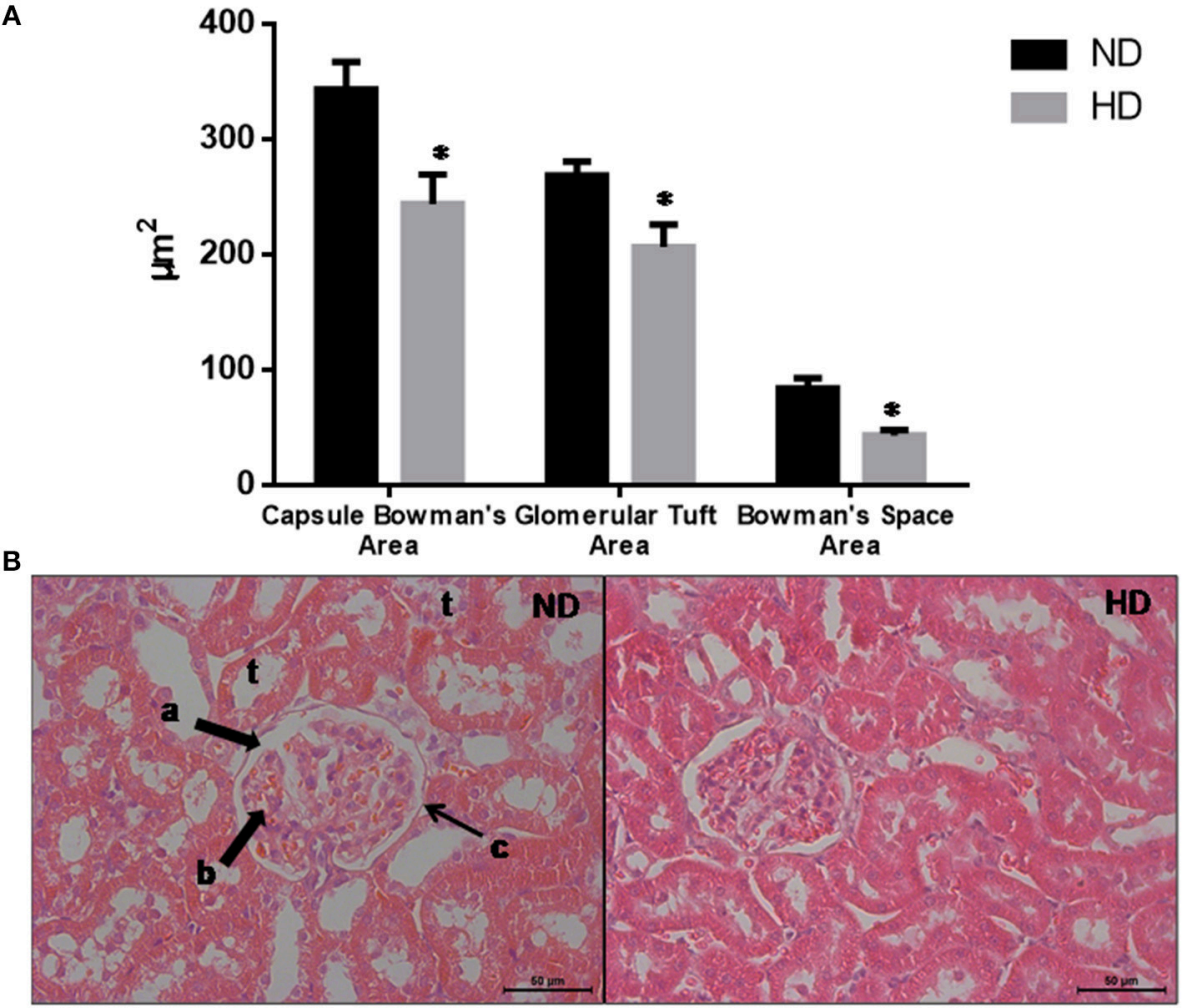
Glomerular morphological parameters. **(A)** Morphologic parameters of renal histology. **(B)** Representative glomeruli. Lowercase letters show: a. Bowman's Space; b. glomerular tuft; c. Bowman's capsule; t: tubules. Magnification: X400. ND, Normal Diet; HD, High-Fat Diet. *p ≤ 0.05 vs. ND.

The morphometric measurements evaluated demonstrate that the glomeruli were retracted in the HD kidneys as shown by the reduction of the BCA (30%), GTA (27%), and BSA (49%) (Figure 2).

**Figure 3:**
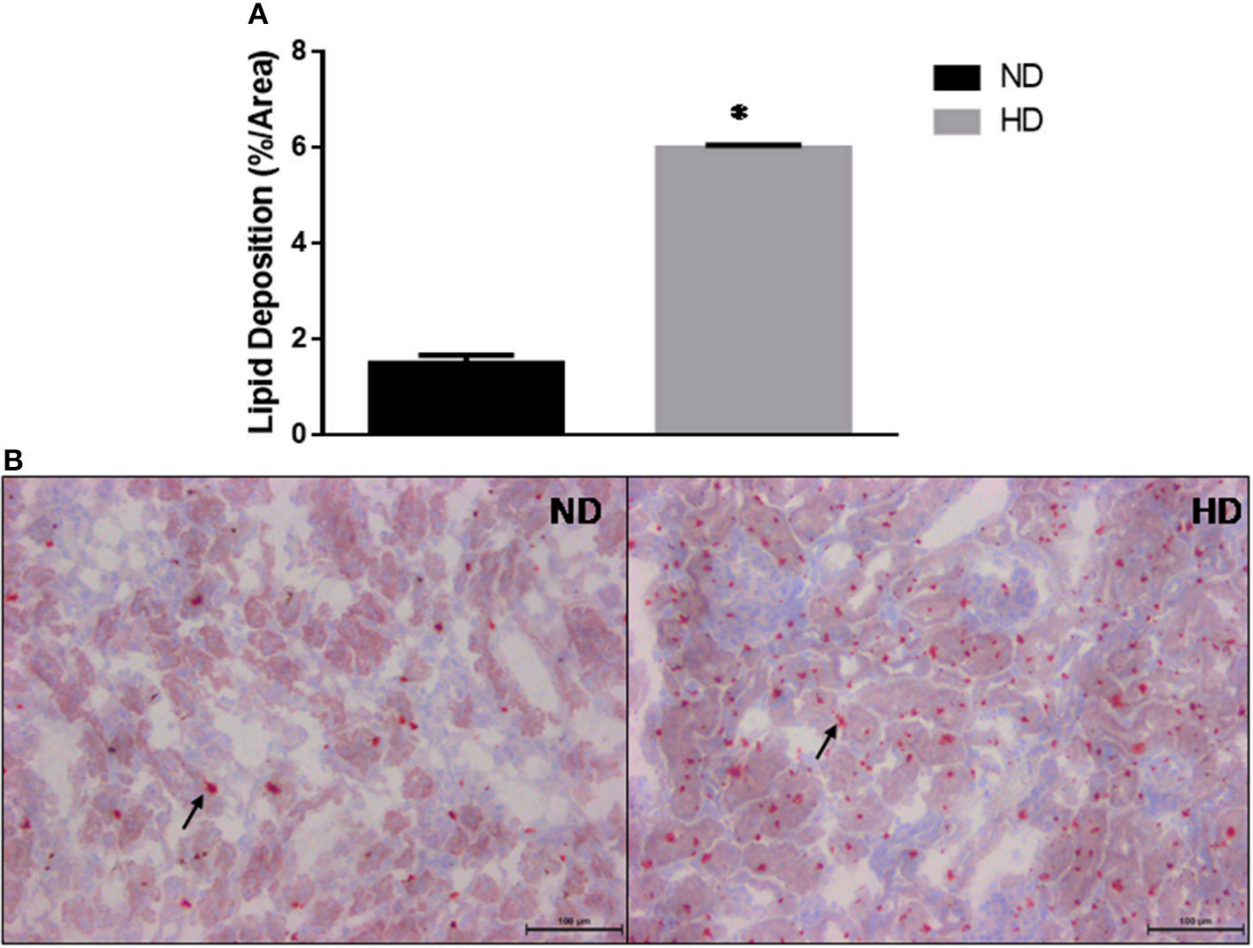
Kidney lipid deposition. **(A)** Estimated percentage of lipids in the kidney. **(B)** Lipid deposition in ND and HD, arrows show lipids droplets. Magnification 200X. ND, Normal Diet; HD, High-Fat Diet. *p ≤ 0.05 vs. ND.

The kidney lipid deposition showed a 3-fold increase in HD compared to ND (ND: 1.5 ± 0.17%/area; HD: 5.9 ± 0.06%/area) (Figure 3).

### Inflammatory Markers

**Figure 4:**
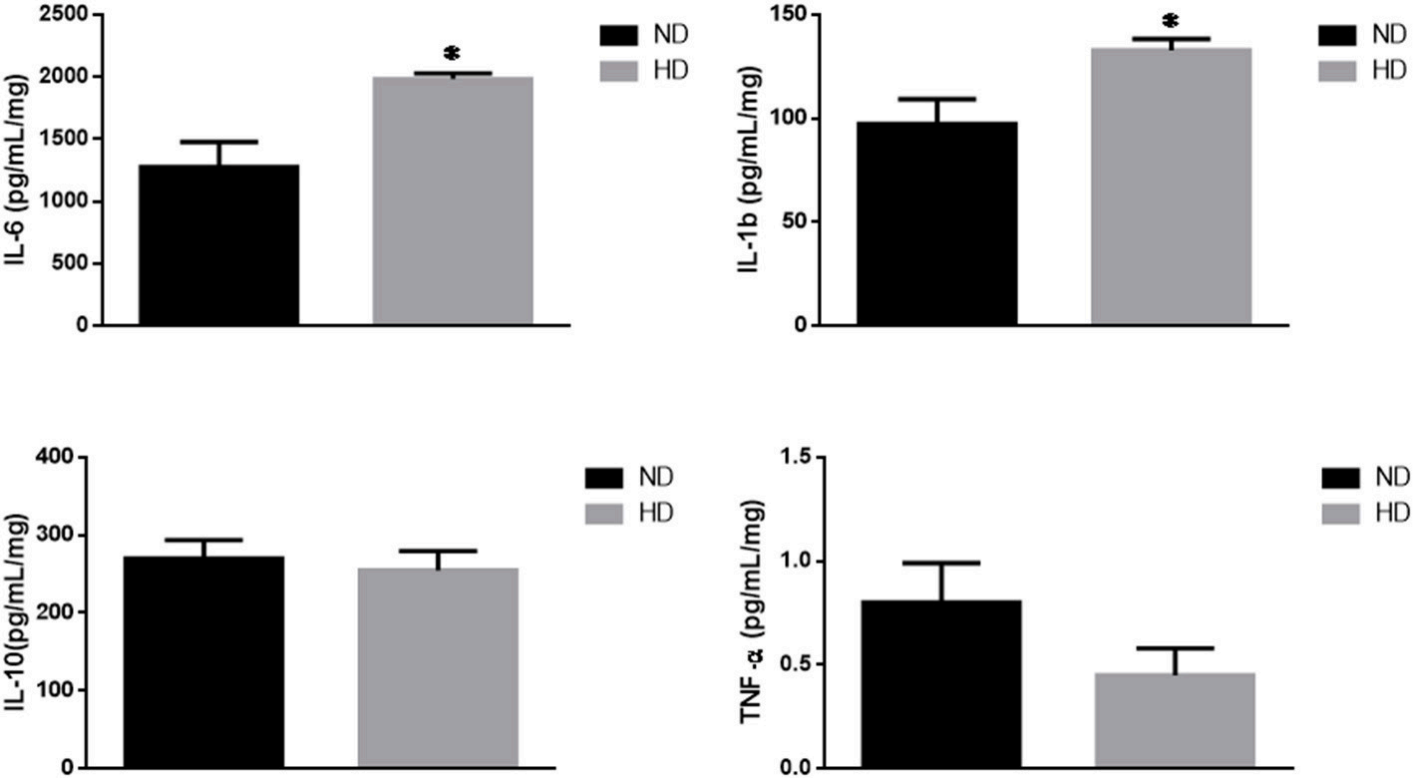
Inflammatory cytokines. Effects of high-fat diet on cytokines (pg/mg of protein): **(A)** Interleukin 6 (IL-6), **(B)** Interleukin 1b (IL-1b), **(C)** Interleukin 10 (IL-10), and **(D)** Tumor Necrosis Factor-alpha (TNF-α). ND, Normal Diet; HD, High-Fat Diet. *p ≤ 0.05 vs. ND.

IL-6, a pro-inflammatory cytokine, was significantly increased in HD (ND: 1,276 ± 203; HD: 1,982 ± 47 pg/mg of protein). In addition, IL1b was also increased (ND: 97 ± 12 HD: 133 ± 5 pg/mg of protein), with no remarkable changes in the TNF-α and IL-10 levels (Figure 4).

### Correlations Analysis

We observed a strong negative correlation between intra-renal lipid content and GFR (*R* = −0.84, *p* = 0.0097). Furthermore, there was a positive correlation between intrarenal lipid content and IL-6 (*R* = 0.79. *p* = 0.02).

## Discussion

In the present study, we investigated the morphological and functional kidney responses in adult rats, exposed to a high-fat diet after weaning. Our findings provide evidence that the consumption of this diet during this critical developmental period induces renal lipid accumulation, increases pro-inflammatory cytokines content and loss of renal function.

After 8 weeks of high-fat diet, the HD group presented no difference in weight gain, and a reduction in food but not at caloric consumption compared to the ND group. These data corroborate our previous demonstration that rats fed a high-fat diet consume less food and have no increase in body weight gain, but have significantly higher adiposity (11). In addition, this animal model of metabolic syndrome is characterized by increased leptin and triglycerides levels, but lower adiponectin and normal insulin levels (11). In this context, we decided to investigate the potential renal complications associated with the current model.

We observed that HD animals did not increase the fasting blood glucose, but presented an increase in AUC and a decreased Kitt. These results demonstrate that these animals developed glucose intolerance and insulin resistance. These results were similar to those of who demonstrated the same response in mice fed a cafeteria diet (32).

The high-fat diet intake induced *in vivo* changes in renal function, as observed by the decrease in GFR and serum creatinine accumulation (33, 34). Moreover, in the isolated perfused kidney we also observed a decrease in GFR. Interestingly, the animals fed a high-fat diet had an increased intra-renal lipid content. Our study corroborates Muller et al. (20) which observed a higher kidney lipid content in mice fed a cafeteria diet. Similarly, Bobulescu et al. (27) showed in humans, a direct association between body mass index and kidney lipid deposition. Kidney lipid accumulation has been associated with renal function injury and can be a risk factor in CKD (19). However, little is known about how this pathogenic process occurs in the kidneys, especially when compared to the knowledge base regarding the deleterious effects of lipids on other organs such as the heart, liver, and skeletal muscle (35).

The intrarenal lipid accumulation is correlated to the GFR reduction in the HD group and can be the cause of morphological alteration in the glomeruli observed by a decrease in the Bowman's capsule, Bowman's space and GTAs demonstrating a glomeruli retraction. In a previous study from our group, Muller and coworkers showed that mice fed a cafeteria diet presented similar morphological kidney damage (20). These glomeruli retraction could be at least in part induced by mesangial cell contraction (MCC). It was demonstrated that MCC could be induced by release of vasoactive hormones such as angiotensin II, that decrease the capillary surface area and consequently reducing the GFR (36–40).

To support this idea, recently our group showed in isolated perfused kidneys obtained from rats under high fructose diet, another experimental metabolic syndrome model, a progressive fall in the GFR associated by an increase in the renal concentrations of angiotensin I and angiotensin II (41). Moreover, unpublished data from our laboratory showed that angiotensin II blockade by losartan in the isolated perfused kidney method determine a higher RVR decrease more in the HD group than the ND group suggesting that the angiotensin II has an important contribution to the RVR rise in the HD.

In the present study, the isolated perfused kidney also demonstrated that in HD group there is an increase in RVR with no changes in tubular sodium and potassium transport suggesting that changes in renal function are associated with glomerular alterations.

We have also shown that the inflammatory markers were changed by the high-fat diet consumption, as observed by the increase in the kidney pro-inflammatory cytokines IL-6 and IL-1b. Interestingly there was no change in the amount of the anti-inflammatory cytokine IL-10. Considering that we did not measure macrophages infiltration in the kidney, we cannot affirm if the source of cytokine production is local or from other tissues, such as adipose tissue. Despite this limitation, it is important to consider that the increased concentration of pro-inflammatory cytokines in the kidney reveals that this tissue is exposed to the deleterious effects typically generated by chronic inflammation, and therefore, may increase the risk of development of lipotoxicity and CKD. Additionally, we observed a positive correlation between kidney lipid accumulation and the IL-6 content in this organ. Saja et al. (42) have demonstrated that animals with dyslipidemia developed inflammation that played a key role in mediating the deleterious changes in kidney function. Previous experiments have also shown a strong association between renal lipid accumulation and increased renal pro-inflammatory mediators, such as interleukin-1 (IL-1), interleukin-6 (IL-6), and TNFα (19). The renal lipotoxicity is also strongly associated with the development of proteinuria, glomerulonephritis and CKD (27). Moreover, Chung et al. (43) have demonstrated that hypertensive animals fed a high-fat diet have increased the angiotensin II, which is associated with kidney lipid deposition, lipotoxicity, and inflammation (43). In this context, our results suggest that the loss of renal function in the HD group can be caused by a lipotoxicity process however more experiments are necessary for the future to support this idea.

## Conclusion

Our study provides evidence that high-fat diet consumption leads to renal lipid accumulation, increases inflammatory cytokines, induces glomeruli retraction, and renal dysfunction. These damages observed in the kidney could be associated with an increased risk to advanced CKD in adulthood suggesting that reduction of high-fat ingestion during an early period of life can prevent metabolic disturbances and renal lipotoxicity.

## Ethics Statement

This study was carried out in accordance with the recommendations of Guide for the Care and Use of Laboratory Animals published by the US National Institutes of Health (n. 85-23, revised in 1996) and approved by the Ethics Committee of Mackenzie Presbyterian University (Protocol: 063/02/2010).

## Author Contributions

CM participated in research design, writing of the paper, performance of the research, and data analysis. AL and RP participated in the performance of research, data analysis and writing of the paper. RY and AA participated in the performance of research and data analysis. NN participated in the performance of research. FE, VF, PF, and MF participated in the performance of research and writing of the paper.

### Conflict of Interest Statement

The authors declare that the research was conducted in the absence of any commercial or financial relationships that could be construed as a potential conflict of interest.

## Abbreviations

MCC: Mesangial cell contraction
HD: High-fat diet
ND: Standard normal diet.
